# Complete Genome Sequence of *Escherichia coli* Phage APC_JM3.2 Isolated from a Chicken Cecum

**DOI:** 10.1128/genomeA.01292-17

**Published:** 2018-01-04

**Authors:** Jayne Mullally, Stephen R. Stockdale, Muireann K. Smith, Lorraine A. Draper, Paul R. Ross, Colin Hill

**Affiliations:** a School of Microbiology, University College Cork, Co. Cork, Ireland; b APC Microbiome Institute, Biological Sciences Institute, University College Cork, Co. Cork, Ireland; c Teagasc Food Research Centre, Moorepark, Fermoy, Co. Cork, Ireland

## Abstract

Avian pathogenic *Escherichia coli* (APEC) bacteria are a significant challenge to the poultry industry. Bacteriophages (phages) have the potential to control APEC strains, increasing animal welfare and economic productivity. Here, we report the isolation of an *E. coli*-infecting phage, APC_JM3.2, isolated from the cecum of a broiler chicken in Ireland.

## GENOME ANNOUNCEMENT

In this study, the bacteriophage APC_JM3.2 was isolated from the cecum of a broiler chicken in Ireland. Phage APC_JM3.2 was detected using the bacterium *Escherichia coli* strain JM3.2 as its host, which was also isolated from the same chicken cecum sample. Virulence factors associated with *E. coli* JM3.2, and its classification as an APEC strain, were not investigated in this study.

The morphology of phage APC_JM3.2 was determined by transmission electron microscopy (UCD Conway Institute of Biomolecular and Biomedical Research, Dublin). The short noncontractile tail of phage APC_JM3.2 indicates that it belongs to the *Podoviridae* family. Biological characterization of phage APC_JM3.2 revealed that it has a latent period of between 15 and 20 min and a burst size of approximately 45 phages per infected cell.

The APC_JM3.2 phage genome was sequenced by using Illumina MiSeq technology (Teagasc Food Research Centre, Moorepark, Fermoy, Co. Cork). Reads were trimmed and filtered by using Trimmomatic (1) to remove adaptor sequences and reads less than 70 bp when a sliding window of 4 bp and minimum Phred score of 30 was applied. Subsequent read quality was assessed using FastQC (http://www.bioinformatics.babraham.ac.uk/projects/fastqc), and assembly was performed using SPAdes Genome Assembler v3.9.0 (2). An average genome coverage of 1,254× for phage APC_JM3.2 was achieved. Putative phage coding sequences were identified using Prodigal v1.20 (3), and functions were predicted by querying translated nucleotide sequences against the BLAST nr and UniProt TrEMBL databases. No tRNA- or transfer-messenger RNA (tmRNA)-encoding sequences could be detected using ARAGORN v1.2.36 (4).

Phage APC_JM3.2 has a 39,761-bp double-stranded DNA (dsDNA) genome with a GC content of 47.3%. APC_JM3.2 is predicted to encode 57 open reading frames (ORFs), which translate into proteins ranging in size from 37 to 911 amino acids. Thirty-five of the APC_JM3.2 ORFs are predicted on the forward strand, while the remaining 22 ORFs have a reverse orientation. Forty-three ORFs of APC_JM3.2 were assigned putative functions by BLASTp, while 11 of the remaining 14 ORFs not assigned a BLASTp function were assigned a putative function by the UniProt database.

The identification of an integrase on the genome of phage APC_JM3.2 indicates that it can lysogenize target bacteria, limiting its usefulness in the control of APEC strains. However, phage APC_JM3.2 is dissimilar to other known *E. coli* phages, with 96% identity across only 47% of its genome to its closest detected BLASTn search result, *Enterobacteria* phage IME10. Therefore, the screening of chicken ceca should yield more phages that are genetically distinct and have therapeutic potential against bacterial infections caused by avian pathogenic *E. coli*.

### Accession number(s).

The complete genome sequence of *Escherichia coli* phage APC_JM3.2 has been deposited in GenBank under the accession number MG197996.
